# ﻿Investigating a hybrid mixed population leads to recognizing a new species of *Arctostaphylos* (Ericaceae)

**DOI:** 10.3897/phytokeys.251.139172

**Published:** 2025-01-16

**Authors:** Tito Abbo, Morgan A. Stickrod, Alexander Krohn, V. Thomas Parker, Michael C. Vasey, William Waycott, Amy Litt

**Affiliations:** 1 University of California, Riverside, USA University of California Riverside United States of America; 2 San Francisco State University, San Francisco, USA San Francisco State University San Francisco United States of America; 3 Tangled Bank Conservation, Asheville, USA Tangled Bank Conservation Asheville United States of America; 4 Nipomo Native Seed, Nipomo, USA Nipomo Native Seed Nipomo United States of America

**Keywords:** *Arctostaphylos*, conservation, ddRADseq, Ericaceae, fragmented population, hybridization, new species, reproductive isolation

## Abstract

While investigating the potential for *Arctostaphylos* species to hybridize in the mixed populations of Point Sal and Burton Mesa in Santa Barbara County, California, we discovered that *Arctostaphylos* from the Nipomo Mesa (San Luis Obispo County), formerly considered a northern population of *A.rudis*, are genetically and morphologically distinct. We name this new taxon *A.nipumu* after the ytt (Northern Chumash language) word for the Nipomo Mesa region. For morphological and molecular analyses, we sampled 54 plants, focusing on *A.purissima*, *A.rudis*, and *A.crustacea* from multiple species and comparative single species populations. Parametric and nonparametric clustering analyses (STRUCTURE and PCA) of ddRADseq data show that *Arctostaphylos* from the Nipomo Mesa segregate from all other samples in the dataset. In mixed populations *A.purissima* and *A.crustacea* samples cluster with samples from other unmixed populations of the same species but *A.rudis* samples form two distinct clusters. One is composed of the mixed populations in Santa Barbara County, and the other consists of the Nipomo Mesa population. Additionally, the Santa Barbara County *A.rudis* samples are admixed in STRUCTURE analysis unlike the samples from the Nipomo Mesa. A principal component analysis of eight morphological characters shows that *A.rudis* individuals from Santa Barbara County tend to be phenotypically variable, occurring in a wide morphological cluster that overlaps with the tight clusters formed by *A.purissima*, *A.crustacea*, and *Arctostaphylos* from the Nipomo Mesa. Based on this evidence we describe the Nipomo Mesapopulation as a new species of *Arctostaphylos*. Given its limited and fragmented distribution we believe that *A.nipumu* is of critical conservation concern.

## ﻿Introduction

*Arctostaphylos* Adans. species in California have a potential to hybridize and there are multiple tetraploid taxa that are thought to have arisen from a hybrid origin (Wells 2000; Parker and Vasey 2016; Parker et al. 2020). It is therefore not surprising to find individuals with intermediate traits in a stand of multiple *Arctostaphylos* species. By the same token, it is surprising that *Arctostaphylos* species can remain distinct despite frequently occurring in mixed stands. Parker et al. (2020) studied pairs of sympatric *Arctostaphylos* species throughout California and made two key observations. First, they noticed that a significant portion of sympatric pairs were composed of a diploid and a tetraploid *Arctostaphylos* species. This suggested that one mechanism of reproductive isolation was reduced meiotic compatibility for interploidal crosses. Out of the currently recognized 108 *Arctostaphylos* taxa, 68 (63%) are diploid taxa while 40 (37%) are tetraploid. The second key observation by Parker et al. was that the majority of sympatric diploid pairs were composed of *Arctostaphylos* species from different major ITS lineages. Molecular phylogenies of the Internal Transcribed Spacer (ITS)-locus revealed two major lineages of *Arctostaphylos*, the large ITS-clade containing ca. 78% of the diploid *Arctostaphylos* species, and the small ITS-clade containing the remaining ca. 22% diploid species (Markos et al. 1998; Boykin et al. 2005; Wahlert et al. 2009). Parker et al. also observed that the subset of sympatric pairs of *Arctostaphylos* species from the same ITS-clade were far more likely to include putative hybrid, morphologically intermediate individuals compared to sympatric pairs of *Arctostaphylos* species from different ITS-clades. This suggested that at least some reproductive isolation had formed between the two clades (hereafter the small and large clades). Thus, they hypothesized that the two primary reproductive isolation mechanisms in *Arctostaphylos* are isolation by genetic distance (ITS-clade incompatibility), and meiotic incompatibility between species of different ploidies. Although beyond the scope of this study, it should be noted that taxon-specific ploidy determination in *Arctostaphylos* has been based on chromosome counts of very few, generally single, individuals (see e.g. Wells 1968) and with the exception of Schierenbeck et al. (1992), there are no broad scale within taxon/population cytological studies in the genus. Therefore, the typical effects of hybridization on ploidy are unknown in *Arctostaphylos*.

**Figure 1.**
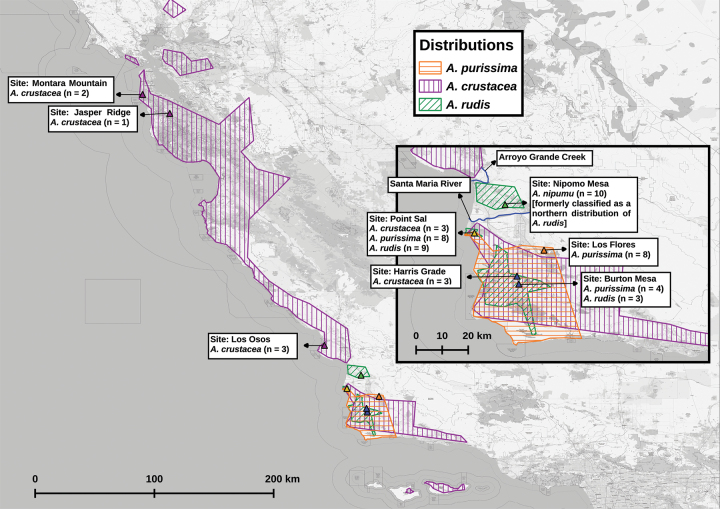
Distribution and sampling map generated in QGIS version 3.224. The background map of the central California coastline is from OpenStreetMap using the EPSG:3857 - WGS 84 / Pseudo-Mercator projection. The distributions of *Arctostaphylospurissima* (orange polygon), *A.crustacea* (purple polygon), and *A.rudis* (green polygon) were inferred using collection data from the Consortium of California Herbaria (CCH2 Portal 2024). The locations where we obtained samples for this study are marked with solid triangles, and site names, taxa sampled, and number of samples obtained for each site are annotated on the figure. The inset shows a closer view of the Nipomo Mesa and northern Santa Barbara County, and the key waterways of the Arroyo Grande Creek, and the Santa Maria River are highlighted in blue. Note that the northern green polygon was formerly considered a northern extension of *A.rudis* but herein is described as the new species *A.nipumu*.

The Central Coast of California is ideal for investigating interspecific gene flow in *Arctostaphylos*. This region hosts the largest number of taxa in *Arctostaphylos* (Cody 1986; Keeley 1992; Vasey and Parker 2014, Burge et al. 2016), especially local endemics, likely reflecting considerable edaphic diversity and a strong coast-to-inland temperature and precipitation gradient (Vasey et al. 2014). Further, stands of *Arctostaphylos* along the coast frequently contain multiple species. We investigated one such stand from central California, Point Sal in Santa Barbara County (Fig. 1), containing two narrow endemic species, *A.rudis* and *A.purissima*, as well as a widespread species, *A.crustacea*. The endemic species in our study are generally restricted to sandy soils under a marine fog-moderated climate (Richerson and Lum 1980; Vasey et al. 2012, 2014) and are of conservation concern (CNPS 1.B.1 or 1.B.2).

The two narrow endemics are diploids and are from different ITS-clades, while *A.crustacea* is a tetraploid from the large clade (Wahlert et al. 2009), so we expected only limited gene flow. Despite these differences in likely ploidy and ITS-clade, a number of individuals at the site exhibited intermediate morphologies, indicating potential hybridization. Nevertheless, we hypothesized that we would find only limited gene flow among all these species in the mixed population. We tested our expectations using genomic data to look for signatures of hybridization and assess if these varied between populations. During this investigation, we inadvertently learned that individuals currently classified as *A.rudis* varied morphologically and genetically among sites. We found that the type locality for *A.rudis*, Point Sal (Jepson 1938), as well as another population farther south, are part of an extensive hybrid zone. The *Arctostaphylos* population from the Nipomo Mesa, north of the Santa Maria River, segregated genetically and morphologically from these hybrid individuals to the south of the river. Here we present the evidence for hybridization, and recognize the distinct Nipomo Mesa population as a new species.

## ﻿Methods

### ﻿Sampling

This study included 54 samples, summarized in Fig. 1. All samples were from mature, adult shrubs. Our goal was to test the prediction of Parker et al. (2020) that species of differing ploidy and/or ITS-clades in a mixed population of *Arctostaphylos* would not exhibit interspecific gene flow. Therefore, Point Sal (Fig. 1, yellow triangle), located at the northern tip of Santa Barbara County California, was our primary study site because *A.crustacea* (tetraploid, large clade), *A.purissima* (diploid, large clade), and *A.rudis* (diploid, small clade) occur there in sympatry. At Point Sal, we collected samples from three individuals of *A.crustacea*, eight individuals of *A.purissima*, and nine individuals of *A.rudis*. The Burton Mesa and Harris Grade sites (Fig. 1, blue triangles), located near Lompoc in Santa Barbara County, together serve as a pseudoreplicate of Point Sal because *A.purissima* and *A.rudis* occur there in sympatry, and a population of *A.crustacea* is close by. We collected samples from three individuals of *A.rudis*, and four individuals of *A.purissima*, at Burton Mesa and three individuals of *A.crustacea* at Harris Grade (ca. 5 Km from Burton Mesa). We selected the remaining sampling localities based on the distributions of *A.crustacea*, *A.purissima*, and *A.rudis* (Fig. 1 polygons), which we estimated using herbarium collection records from the California Consortium for Herbaria (CCH2 Portal 2024). We used these distribution maps to devise a sampling scheme which accounted for both the ranges of our focal taxa and the regions where these ranges did not overlap. To account for potential genetic changes across its wide range, we split the remaining sampling of *A.crustacea* between the northern and southern edges of its core range. We collected samples from three individuals at the Los Osos population in San Luis Obispo County, and three individuals from San Mateo County, one of these from Montara Mountain, and two from the nearby Jasper Ridge. For *A.rudis*, we collected 10 samples from the Nipomo Mesa, a disjunct population in San Luis Obispo County north of Point Sal. Lastly, we sampled from eight individuals of *A.purissima* from Los [sic] Flores Ranch Park in northeastern Santa Barbara County.

For all samples, we froze fresh material of flower buds and/or immature leaves; we stored this tissue at -20 C until DNA extraction. For each sample we also collected a herbarium voucher specimen, dried and preserved for documentation and morphological analysis. We deposited voucher specimens at the UCR Herbarium, and when sufficient excess material was present, we deposited a duplicate at the Harry D. Thiers Herbarium at San Francisco State University. The appended Suppl. material 1 contains further sample information including GPS coordinates and collection numbers.

### ﻿Molecular sample preparation and sequencing

From each sample we extracted DNA using the Qiagen DNeasy Plant mini kit (QIAGEN, Venlo, The Netherlands) following manufacturer’s instructions and confirmed the presence of high molecular weight DNA on a 1% agarose gel. We subsequently used these DNA extracts to prepare ddRADseq libraries. Although we collected the majority of our samples in December, 2023, we collected some of our samples in the years 2018–2020 as part of an ongoing genus-wide, phylogenomic study (Suppl. material 1). For the four samples of *A.purissima* and three samples of *A.rudis* from Burton Mesa only (sample codes: I7, I8, J1, J2, J3, J5, J6; see Suppl. material 1), we used sequences we obtained in 2020 from the UCR Institute for Integrative Genome Biology Genomics Core. We prepared these libraries following the exact protocol published in Huang et al. (2020). We prepared the remaining 47 samples in 2024 using a modified version of the Huang et al. protocol. The main modifications we implemented were: 1) We increased the time that DNA samples were digested with restriction enzymes at 37 C from three hours to six hours. In a set of bench trials, Morrison et al. (unpublished), observed that this increased digestion time seemed to yield a more complete digestion and had no observable down sides. 2) We carried out the size selection by gel excision earlier in the protocol to increase workflow efficiency. Here, we implemented a mini pooling strategy where we combined samples into mini pools of 8–10 samples, size-selected these pools via gel excision, and then combined them into a single pool after PCR amplification. 3) We increased the volume of DNA template solution from 1.25 μl to 2.65 μl per PCR reaction. We kept the volume of all other PCR reagents the same as Huang et al. by not adding additional water to the PCR master mix i.e. the increased DNA template volume corresponded to the amount of water added in the original protocol. Bench trials by Morrison et al. (unpublished) showed that this modification favored the amplification of DNA library material over a competing dimer reaction. 4) We eliminated the Evrogen (Moscow, Russia) Timmer Kit reaction. This Timmer Kit reaction was highly inefficient in terms of DNA recovery and therefore required a second PCR reaction which increased stochasticity of pooled sample amplification. The relatively smooth curve we detected in our Agilent Bioanalyzer (2100 expert High Sensitivity) quality control showed that it was unnecessary. These modifications improve the consistency of successful library preparation and the resulting sequence data remains compatible with batches prepared using the original Huang et al. (2020) methods. Abbo (unpublished) combined a larger set of data prepared with both methods and did not observe any batch effects. We then submitted the finished libraries to GeneWiz (Azenta, New Jersey, USA) for sequencing on a partial lane of an Illumina NovaSeq sequencer with 150 bp paired-end configuration.

### ﻿Molecular data analysis

We quality controlled and aligned the raw sequence reads to the genome of *Arctostaphylosglauca* (Huang et al. 2022) using IPYRAD version 0.9.95 (Eaton and Overcast 2020). We kept parameters at default for paired ddRADsequencing reads with the exception of the “min_samples_locus” parameter which we set to 15, corresponding to ca. 28% of the total number of ingroup samples. Wagner et al. (2018) tested the sensitivity of inferred phylogenetic topology to changes in the “min_samples_locus” parameter and found that the extremely low default value of four or an extremely high value corresponding to 100% of samples in the dataset yielded unreliable results, and intermediate values only affected support values and not recovered topology. Consistent with Wagner et al.’s results, preliminary assemblies of other *Arctostaphylos* datasets showed that values greater than 25% maximize the amount of data without harming the reliability (Abbo, unpublished). We used the default maximum number of alleles in the consensus sequence as two despite having tetraploids in our dataset. Huang et al. (2020), using our same sequencing method, found equivalent results when treating tetraploid *Arctostaphylos* samples as diploid in the context of maximum number of alleles and recommended this approach for future studies in the genus. Burge et al. (2018) also used two as their maximum number of alleles for a dataset of tetraploid *Arctostaphylos* samples using single digest RAD-sequencing.

A key difference between these previous studies and the current one is that Huang et al. and Burge et al. only examined one tetraploid *Arctostaphylos* species and we examined diploid *Arctostaphylos* species in combination with one tetraploid species. To validate using two as the maximum number of alleles in the consensus sequence (MA), we tested the effect of setting the MA to four on principal component analysis (PCA). This was not a perfect solution as IPYRAD still assumes diploidy for the purpose of heterozygous base calling when the MA is set to four, but our approach was consistent with other studies that used IPYRAD to process multiple-ploidy datasets (Wagner et al. 2020; Suissa et al. 2022). PCA results were consistent between the two and four MA datasets (Suppl. material 2); however, the issue described below of samples with higher missing data influencing PC1 was more severe in the four MA dataset (Suppl. material 2: fig. S1B). The results were consistent between both datasets when we used K-means missing data imputation (Suppl. material 2: fig. S1A, D) and when we did not use imputation but excluded the four samples with the highest amounts of missing data (Suppl. material 2: fig. S1C, F). When we did not exclude samples nor impute missing data, both datasets showed similar results for PC0, but PC1 showed some informative clustering despite samples with higher missing data plotting as outliers in the two MA dataset, whereas PC1 appeared to only give information about sample-specific missing data in the four MA dataset (Suppl. material 2: fig. S1B, E). We found that the similar PCA results between the two MA and four MA datasets justified using the two MA dataset for STRUCTURE analysis, which produced results that agreed with PCA. All analyses depicted in the main text, herein, were generated using the two MA dataset.

We analyzed the aligned sequences using clustering analyses. We carried out nonparametric clustering as a PCA using the .pca function from the IPYRAD analysis tools (IPA). First, we ran .pca with single nucleotide polymorphisms (SNPs) present in at least 80% of individuals (minimum coverage = 0.8) and with no missing data imputation over 50 replicate runs, each with a different random draw of uncorrelated SNPs. The data separated into four clusters but with four samples with high missing data plotting as outliers. Therefore, we reran the analysis using the same minimum coverage and number of replicates but with K-means missing data imputation set to K = 4. Imputation refers to the process of inferring missing values in the data using patterns in the remainder of the data. We chose K-means imputation as it inferred the missing values from four clusters naturally present in the data rather than relying on our assumptions about taxonomy or geographic distributions.

We then performed parametric clustering using the STRUCTURE (Pritchard et al. 2000) distribution in the STRUCTURE_THREADER package version 1.3.10 (Pina‐Martins et al. 2017). We used STRUCTURE_THREADER to run multiple replicates at various K values simultaneously. To meet the assumptions in STRUCTURE of no linkage between sites, we used the .ustr file generated by IPYRAD from randomly selected unlinked SNPs as the input data file. We used standard parameters in STRUCTURE with admixture set to true, and supplied no population priors to avoid biasing the inferred groups with taxonomic and/or spatial assumptions. We ran STRUCTURE from K = 1 up to K = 10 with three replicates per run (30 runs total), each for 50,000 MCMC generations with the first 10,000 generations omitted as burn-in. We selected these MCMC chain and burn-in lengths based on Burge et al. (2018); in the past we have also experimented with longer MCMC chains in *Arctostaphylos* (Abbo, unpublished) and did not notice changes to the results. We evaluated the best number of K ancestral groups by examining the changes in mean log-likelihood and changes in ΔK across each value of K *i.e.* the Evanno method (Evanno et al. 2005) in R version 4.4.1 using the values calculated by STRUCTURE HARVESTER (Earl and vonHoldt 2012). K = 2 was clearly best supported and the results for higher values of K were virtually identical to those of K = 2 with additional ancestral groups represented only by negligibly low percentages (Suppl. material 3).

### ﻿Morphological data collection and analysis

We analyzed morphological variability across species and populations in the data by measuring eight morphological characters, available for all samples; for each individual, we recorded traits in the field or from their voucher specimens after collection. The characters we used were: 1) Estimated height of individual (continuous): the height of the individual from the ground to the tallest part of the canopy. This was estimated and recorded in the field, in meters. 2) Estimated width of individual (continuous): the length of the apparent widest portion of the individual’s canopy. We estimated and recorded this in the field, in meters. 3) Presence or absence of a burl (categorical): a burl is a woody organ, containing meristematic tissue that develops at the base of the plant and provides a mechanism for regeneration after damage, especially fire. We recorded the presence or absence of this character in the field and numerically coded it as 0 (absent), and 1 (present). 4) Leaves isofacial or bifacial (categorical): leaves with similar color, hairiness, and presence of stomata on both leaf surfaces are considered isofacial, and leaves with different colors or hairiness on leaf surfaces and/or lacking stomata on one surface are considered bifacial. We assessed this character by examining leaves from voucher specimens under a dissecting microscope and numerically coded it as 0 (bifacial), and 1 (isofacial). 5) Twig hair length (categorical): the general trend of length of trichomes on young twigs in three binned categories, short (0–2 mm), intermediate (2–6 mm) and long (>6 mm). We assessed this character by examining young twigs from voucher specimens under a dissecting microscope and numerically coded it as 1 (short), 1.5 (intermediate), and 2 (long). 6) Length of the longest leaf blade (continuous): the length of the blade of the longest leaf on a voucher specimen, which we measured from the end of the petiole to the end of the blade to the nearest tenth of a centimeter. If the leaf was basally lobed or auriculate, we measured the midrib length instead. 7) Length of the petiole (continuous): the length of the petiole of the longest leaf on a voucher specimen, which we measured from the stem to the start of the blade to the nearest millimeter. 8) Leaf base shape (categorical): the general trend of the shape of the base of the leaves on a voucher specimen. We coded this character as 0 (lobed), 1 (truncate), 2 (rounded), 3 (tapered). We coded categorical variables numerically in order to examine all variables using a PCA, which we generated in R version 4.4.1 using the prcomp function with both scale and center set to true.

### ﻿Field surveys for new species description

Because the data support the *Arctostaphylos* population from the Nipomo Mesa as a new species, we carried out additional surveys throughout the Nipomo Mesa to assess its range. For each population that we found we collected a paratype voucher specimen, and we documented morphological characters with field notes and photographs (see Suppl. material 1). We then supplemented these field surveys with satellite data using ArcGIS Pro version 3.3.2 (Esri Inc 2024). We estimated the range and land occupied by *Arctostaphylos* from the Nipomo Mesa (Fig. 5), by converting coordinate data for individual occurrences of plants to point data shapefiles, and we mapped these using the NAD 1983 (2011) StatePlane California V FIPS 0405 (US Feet) coordinate system. We calculated population surface area in hectares by loosely defining population boundaries surrounding single individual occurrences and clusters of individuals within a contiguous area of associated habitat. We then calculated the surface area of the resulting polygons using ArcGIS Pro. Lastly, because the field surveys determined that the individuals from Nipomo Mesa lacked a basal burl, we performed an additional survey at Burton Mesa to confirm that the presence of a burl was not a mistake in the original description of *A.rudis*.

## ﻿Results

### ﻿Genetic analysis

**Figure 2.**
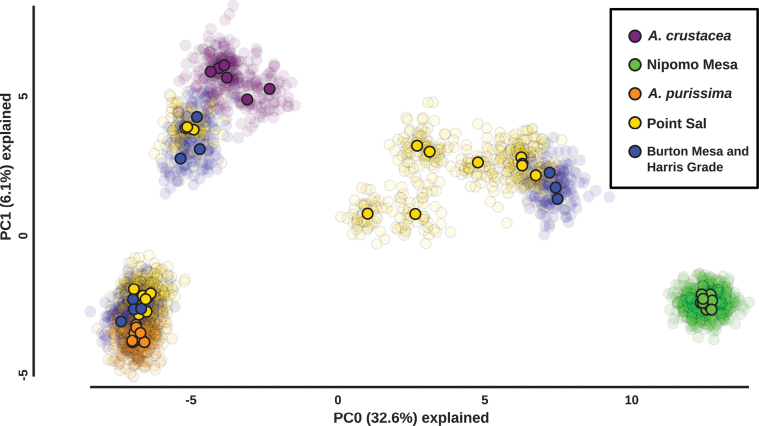
Principal component analysis (PCA) of ddRADseq data with SNPs present in at least 80% of individuals (minimum coverage = 0.8). The figure was generated using the IPYRAD analysis tools (IPYRAD version 0.9.95) and edited in Inkscape version 1.1.2. PC0 is given in the x axis and explains 32.6% of variation in the data, while PC1 in the y axis explains 6.1% of variation in the data. Solid circles are the results of the PCA for a random draw of uncorrelated SNPs for each sample, and the transparent circles are the results of 49 additional PCAs for distinct, independent random draws of uncorrelated SNPs. Missing data was imputed using K-means imputation at K = 4. Samples from the Nipomo Mesa (green), Point Sal (yellow), and Burton Mesa and Harris grade (blue) are color-coded by Sampling location, and samples of *Arctostaphylospurissima* (orange), and *A.crustacea* (purple) from control/nonfocal populations are color-coded by taxonomic identity.

Principal component analysis (PCA) reveals four groups of samples. PC0 explains the majority of variation in the data (15% without missing data imputation; > 30% using K-means, K = 4, missing data imputation) and can on its own be used to separate the samples into four clusters, but the two taxa from the large clade, *A.crustacea* and *A.purissima*, are very close together. We observe further separation along PC1, the second axis of variation; however, without imputing missing data, the four samples with the highest amounts of missing data (samples: I8, CZ6 DA3, and DC5; see Suppl. material 1) group on their own along PC1 (see Suppl. material 2: fig. S1E). We observe no PC1 outliers using K-means imputation at K = 4 (Fig. 2). The samples from the Nipomo Mesa and the samples of *A.purissima* form the tight clusters at the opposite bottom corners of the figure. The *A.crustacea* samples form a slightly wider cluster with low values of PC0 and high values of PC1. The fourth and widest cluster consists of 12 samples from Point Sal and Burton Mesa that we morphologically determined as *A.rudis*; this cluster is intermediate to the other three clusters in principal component space. The remaining samples from Point Sal, Burton Mesa and Harris Grade group with either *A.purissima* or *A.crustacea* consistent with the morphological identification of those respective samples. No other samples cluster with the samples from the Nipomo Mesa. The transparent points in Fig. 2 are the results of 50 repeated principal component analyses using a different randomly selected set of unlinked single nucleotide polymorphisms (SNPs). We observe minimal variation among these replicate analyses suggesting that the results are robust.

**Figure 3.**
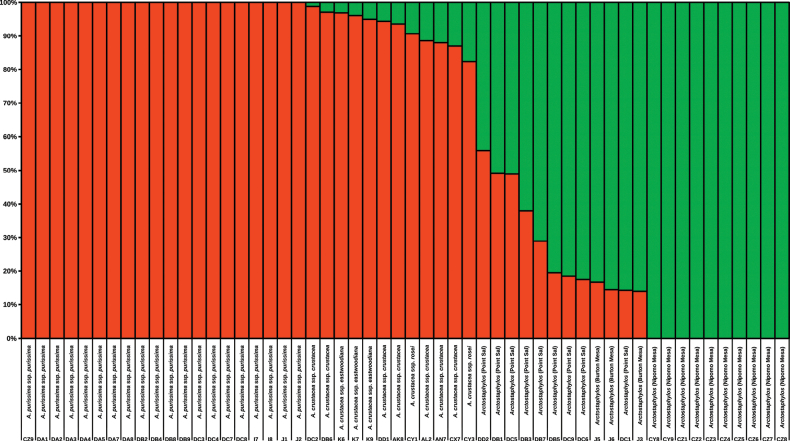
Structure plot/stacked bar graph of the K = 2 using uncorrelated SNPs from ddRADseq data. Structure was run using STRUCTURE_THREADER version 1.3.10 for 50,000 MCMC generations with the first 10,000 discarded as burn-in. The figure was generated using LibreOffice Calc version 7.3.7.2 and edited in Inkscape version 1.1.2. Each individual sample is represented by a unique 1-2 letter and single number code given at the bottom of the x axis. Above the code, we indicated the individual’s source population, or its taxonomic identity for control/nonfocal populations of *Arctostaphylospurissima*, and *A.crustacea* (see Suppl. material 1 for more information about samples). The y axis indicates the genotype percentage of the two ancestral groups (colored as green and orange) inferred for each sample.

The parametric clustering analysis, STRUCTURE, Fig. 3 shows results for all samples at K = 2. Results for higher values of K are not supported, and the additional ancestral groups occur at such low percentages that their plots look practically identical to Fig. 3 (Suppl. material 3). The two K clusters are correlated with major ITS-clade membership. All *A.purissima* (large clade) samples are 100% associated with the same ancestral group, depicted in orange. The *A.crustacea* samples, the other large clade taxon, are mostly composed of the orange ancestral group but always have at least a small amount of admixture from the second ancestral group, depicted in green. The samples from the Nipomo Mesa are entirely associated with the green ancestral group. Although the samples currently classified as *A.rudis* (small clade) are primarily associated with the green ancestral group, the same 12 samples (from Point Sal and Burton Mesa) that are intermediate in the principal component analysis are recovered with substantial admixture from the orange ancestral group.

### ﻿Morphological analysis

**Figure 4.**
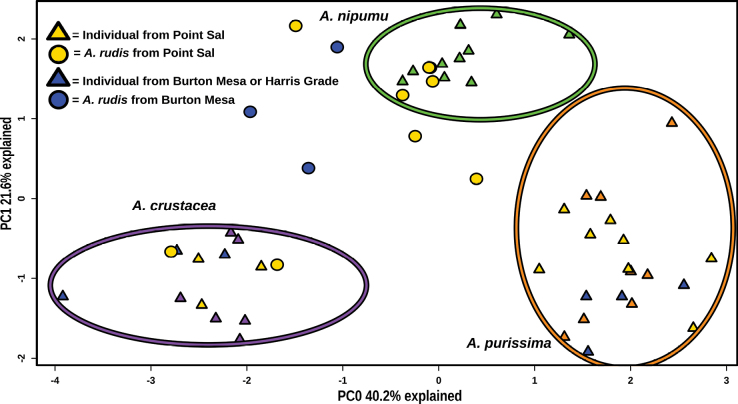
Principal component analysis (PCA) of morphological data. The figure was generated in RStudio version 2024.04.2, Build 764 and was edited in Inkscape version 1.1.2. PC0 is given in the x axis and explains 40.2% of variation in the data, while PC1 in the y axis explains 21.6% of variation in the data. Ellipses were added manually in Inkscape to highlight groups of points that align morphologically with *Arctostaphylosnipumu* (green), *A.crustacea* (purple), *A.purissima* (orange) and were based on the spread of green, purple and orange triangles (representing samples taxonomically identified as *A.nipumu*, *A.crustacea*, and *A.purissima* respectively). Samples from the focal populations of Point Sal (yellow), and Harris Grade and Burton Mesa (blue) are color-coded by their source populations. Yellow and blue triangles are samples of *A.purissima* or *A.crustacea* and have no evidence of hybridization in the molecular data. Yellow and blue circles are samples of *A.rudis* and are hybrids according to molecular data.

The samples cluster morphologically in similar groups to those revealed by genetic analyses. Because all samples were not at the same phenological stage at the time of collection, we focused on a set of continuous and categorical vegetative traits that were available for all samples. Principal component analysis of these data (Fig. 4) reveals a broadly similar pattern to the genomic PCA (Fig. 2). The main distinction from the genetic data is the lack of a discrete cluster of hybrid samples. The data separate into three main morphological groups represented by the ellipses in Fig. 4. *A.crustacea* and *A.purissima* samples separate into two clusters regardless of population of origin, and the third cluster consists of the samples from the Nipomo Mesa. The hybrid samples from Point Sal and Burton Mesa, depicted as yellow and blue circles respectively, have a significant amount of morphological variability. These samples group with either samples from the Nipomo Mesa, *A.crustacea*, or they are intermediate between the three clusters.

**Figure 5.**
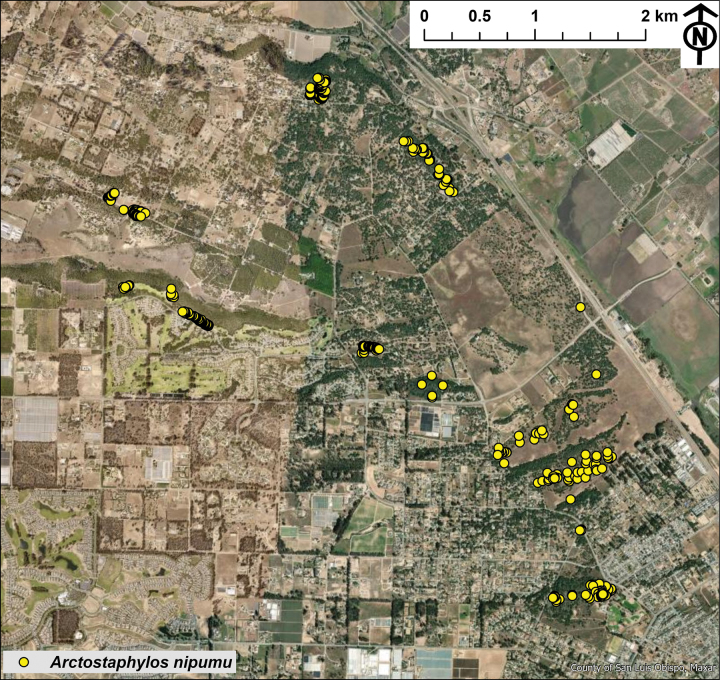
Known occurrences and distribution of *Arctostaphylosnipumu*. Nipomo Mesa, San Luis Obispo County, California. Map figure was produced using ArcGIS Pro 3.3.2 (Esri Inc 2024). Coordinate data for individual occurrences of *A.nipumu* were converted to point data shapefiles and mapped using the NAD 1983 (2011) StatePlane California V FIPS 0405 (US Feet) coordinate system.

We confirmed morphological distinctions between *Arctostaphylos* from the Nipomo Mesa and other populations of *A.rudis* during additional field surveys. At the Nipomo Mesa we found 10 multi-individual occurrences of *A.nipumu* fragmented by urban and agricultural development (Fig. 5). We estimate that these occurrences amount to 300–700 individuals occupying 28.63 hectares of land. Plants from the Nipomo Mesa were readily distinguishable from other *A.rudis* by their lack of basal burls and different bark phenotype. All plants we surveyed at Burton Mesa had basal burls and also have varying degrees of smooth red bark in their younger branches. In contrast, plants from the Nipomo Mesa tended to be erect or layering but lack basal burls and generally have gray shredding bark even in younger branches.

The only exceptions we saw were in the undeveloped areas along the north side of Black Lake Canyon Drive in the vicinity of the Blacklake Golf Resort, which had a mixture of tall individuals lacking burls and with gray, shredding young branches, and short, mounding individuals, with smooth, red young branches and often with large, exposed basal burls. We subsequently verified that that area is a mitigation site required by the County of San Luis Obispo in conjunction with a nearby golf course and housing development (San Luis Obispo County 1982). There was no burn site at the Nipomo Mesa so we could not confirm that *A.nipumu* is unable to sprout after fire. The morphology at the *A.rudis* type locality, Point Sal, is more complex and less consistent than the morphologies observed at the Nipomo Mesa and Burton Mesa, consistent with the higher levels of introgression we detect at Point Sal compared to Burton Mesa (Fig. 3).

## ﻿Discussion

We find unidirectional introgression from the large clade to the small clade at the type locality of *A.rudis* and at Burton Mesa. This is consistent with the hypothesis that some reproductive isolation exists between the three focal species but this isolation is incomplete. All samples of *A.rudis* from these populations form an intermediate cluster in principal component analysis of SNP data (Fig. 2) and have highly to moderately admixed genomes according to STRUCTURE analysis (Fig. 3). Furthermore, these samples are morphologically variable (Fig. 4) as would be expected for early generation hybrids with unequal levels of introgression. Although some signatures of admixture were also detected in *A.crustacea*, these were detected in all sampled populations of this species suggesting this is an older fingerprint of hybridization, more or less stabilized in the genome, and not associated with our focal sites.

Therefore, we conclude that strong signals of hybridization are present in *A.rudis* from Point Sal and Burton Mesa, and this hybridization appears to be associated with multispecies populations because it is absent in the genome of the population from the Nipomo Mesa. Both the morphological and genetic data indicate that the *A.rudis* individuals from Point Sal and Burton Mesa are introgressed with genes from another taxon, potentially *A.crustacea*, or some locally extinct lineage. Meanwhile, the Nipomo Mesa population of *Arctostaphylos* is morphologically and genetically distinct from the other *A.rudis* populations. Because its type locality is Point Sal, the name *A.rudis* refers to hybrid individuals within a hybrid zone. The *Arctostaphylos* species from the Nipomo Mesa is likely one of the parental species of these hybrid individuals but is distinct from, and lacks, the hybrid signal associated with *A.rudis*. We therefore assign this new species the name:

### ﻿ Arctostaphylos nipumu

Taxon classificationPlantaeEricalesEricaceae

T.Abbo, M.A.Stickrod, A.Krohn, V.T.Parker, M.C.Vasey, W.Waycott & A.Litt. sp. nov.

E5DC02C4-EA6E-594C-AC1E-F3C4FCAA175E

urn:lsid:ipni.org:names:77355280-1

Figs 6 , 7

#### Type.

United States of America • California: San Luis Obispo County, Nipomo Regional Park, 200 m southwest (260°) from parking area exit onto Pomeroy Rd., Oceano 7.5’ Quad: 35°01'57.5"N, 120°30'01.8"W, ± 55 m, 114–124 m alt., 28 Dec 2023, *MA Stickrod 135* (***holotype***: UCR!; isotype: SFSU!). ***Paratypes*** (all from San Luis Obispo County, for coordinates and collection dates see Suppl. material 1): • Nipomo Regional Park, MA Stickrod 126 (UCR; SFSU) • same locality, MA Stickrod 128 (UCR; SFSU) • same locality, MA Stickrod 130 (UCR; SFSU) • same locality, MA Stickrod 131 (UCR; SFSU) • same locality, MA Stickrod 134 (UCR; SFSU) • same locality, MA Stickrod 128 (UCR; SFSU) • same locality, T Abbo 147 (UCR; CAS; MO; NY; OBI) • same locality, T Abbo 148 (UCR; DAV; UCSB) • Blacklake, Golf Ball Rd., T Abbo 149 (UCR; IRVC; SBBG; SD) • Blacklake, Black Lake Canyon Dr., T Abbo 150 (UCR) • Los Berros Ridge, Dale Ave., T Abbo 153 (UCR; OBI; RSA) • southeast of Los Berros; Summit Station Rd., T Abbo 154 (UCR; UCSB).

#### Description.

Shrub up to 2, 3 (5) m ht. and < 10 m width; often layering, rooting when branches contact soil; burl 0 but with branchlets sprouting infrequently on stems; bark (red tinged) gray and shredding, from large stems up to new growth; twigs and petioles with moderately to very dense, short, nonglandular hairs; leaves isofacial (with stomata on both surfaces), green, shiny, generally lanceolate to ovate (elliptic to rounded with mucronate tip); blade 1.4–3.2 cm length, 0.9–1.9 cm width; petiole 0.4–0.8 cm; inflorescence a raceme or few-branched panicle (generally < 5-branched), nascent and mature inflorescences of similar length and thickness, 0.5 to 1.1 cm, and ca. 1 mm; bracts ± scale-like, often grading to ± leaf-like proximally, generally green, photosynthetic in summer; flowers 5-merous, urn-shaped, white to pinkish-white; fruit a multi-seeded drupe, generally reddish-orange, depressed axially; mesocarp mealy; endocarp generally rough, fused or separating into a variable number of nutlets.

#### Etymology.

Based on discussions with the yak tityu tityu yak tiłhini (Northern Chumash Tribe), we selected the name *Arctostaphylosnipumu* because nipumu is the ytt (Northern Chumash language) word for the Nipomo Mesa region. The word nipumu is literally translated to English as “of the big house”, so we treat the epithet *nipumu* as a noun in apposition; ergo, *Arctostaphylosnipumu* is translated as “*Arctostaphylos* of the big house”. We recommend that *A.nipumu* be referred to by the common name nipumu manzanita or Nipomo Mesa manzanita; the latter regional name, Nipomo Mesa, is more well known in current usage, but the word Nipomo is an inferior Spanish transliteration of the word nipumu.

#### Distribution.

*Arctostaphylosnipumu* is one of the most narrowly distributed *Arctostaphylos* species, occurring exclusively on Oceano series soil (Soil Survey Staff, USDA) at 100–200 m elevation in the sandy maritime chaparral and adjacent *Quercusagrifolia* woodlands of the Nipomo Mesa. It is the only member of the genus found in this area, and its isolated population appears to be associated with two local waterways, the Santa Maria River to the south, and Arroyo Grande Creek to the north (Fig. 1, inset). Its range is situated within a disjunction in the distribution of *A.crustacea* and just north of the northern edge of the distribution of *A.purissima*. Little is known about the biogeography of *Arctostaphylos* species, so it is unclear what has led to this distribution pattern, which is a topic we are currently investigating.

#### Notes.

*A.nipumu* is most reliably distinguished from the two populations of *A.rudis* using its bark characteristics. *A.nipumu* has gray to reddish-gray bark that shreds in short strips generally all the way to its young branches; the young branches may become progressively redder but the outer bark remains a dull gray or brown that contrasts with the inner bark. The older stem bark of *A.rudis* may appear similar to that of *A.nipumu* but often is redder and shreds in wider strips (Fig. 7), and younger branches tend to be smooth dark red or if shredding, then the outer bark does not contrast with the inner bark. Consistent with its high levels of introgression (Fig. 3), the Point Sal population of *A.rudis* is highly variable. Some individuals may appear very similar to *A.nipumu* but, in addition to having the described bark difference, will be shorter and ± mounded compared to the taller, ± erect *A.nipumu*. Other individuals may be strikingly distinct from *A.nipumu* by having burls, long hairs, and/or truncate to lobed leaves resembling *A.crustacea*, whereas *A.nipumu* lacks a burl, always has short hairs, and typically has lanceolate to ovate leaves (never truncate or lobed). In contrast, *A.rudis* individuals from the Lompoc area (represented in our molecular data by the Burton Mesa population) tend to be more similar to each other as well as to *A.nipumu*. They are, nonetheless, easily distinguishable from *A.nipumu* because they have the pronounced bark difference and consistently have burls. The lack of a burl in *Arctostaphylos* from the Nipomo Mesa was independently observed by Keil and Hoover (2022) who noted in the Vascular Plants of San Luis Obispo County the inconsistency with Jepson’s description of *A.rudis* and the plants on the Nipomo Mesa, writing: “repeated unsuccessful search has been made for the basal burl which is said in the original description to be present. Apparently the presence or absence of that structure is not a distinctive feature of this species.” (p. 448).

**Figure 6.**
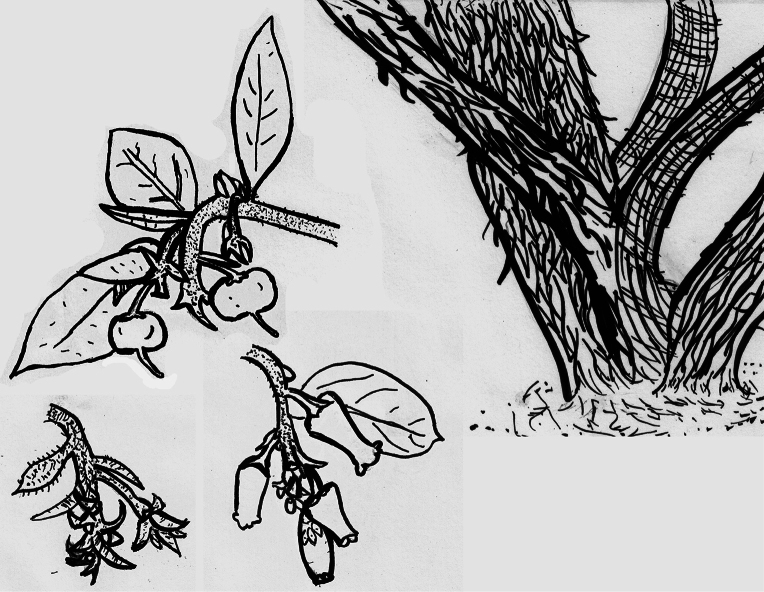
Line drawing of *Arctostaphylosnipumu*. Top right (mature main trunk): bark shredding; base lacking burl. Top left (fruiting branch): twigs and petioles with moderately to very dense, short, nonglandular hairs; leaves generally lanceolate to ovate; fruit depressed. Bottom Left (nascent inflorescences): nascent inflorescences short/compressed; bracts overlapping, ± scale-like. Bottom center (flowering branch): inflorescences short, ± same length as nascent inflorescences; flowers urn-shaped.

**Figure 7.**
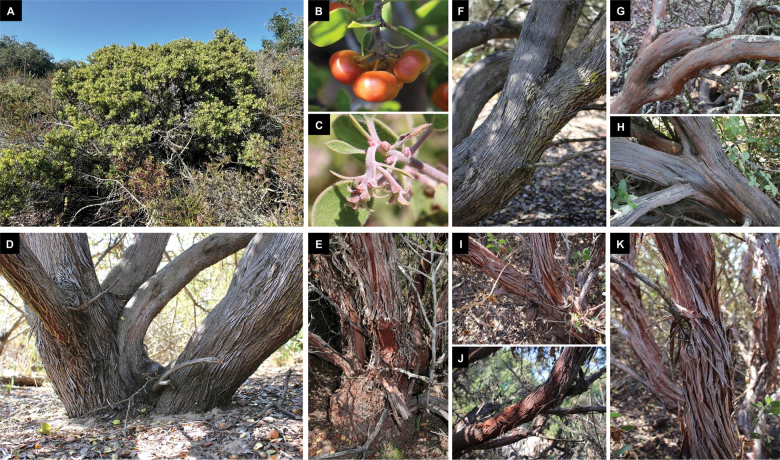
Diagnostic photographs of *Arctostaphylosnipumu* (Nipomo Mesa, San Luis Obispo County, CA) and *A.rudis* (Burton Mesa, Santa Barbara County, CA) **A***A.nipumu* in situ, maritime chaparral habitat **B** depressed fruit of *A.nipumu***C** nascent inflorescence, with leaf-like bracts proximally grading to scale-like bracts distally **D***A.nipumu*, lacking a burl at the base of the stem **E***A.rudis* with a pronounced burl at the base of the stem **F–H** gray to reddish-gray, thinly shredding bark of *A.nipumu***I–K** pronouncedly red to reddish-gray, coarsely and variably shredding bark of *A.rudis*.

While the Point Sal population appears to be composed of recent first and second generation hybrid individuals, the Burton Mesa (Harris Grade south) population of *A.rudis* has lower percentages of admixture (Fig. 3). Currently, we are further investigating the Point Sal and Burton Mesa populations. The questions we are considering include: what is the extent of hybridization among individuals, are these populations genetically stable and uniform, and is monophyly present in a large sample of individuals. For now, we recommend that these populations continue to be referred to under the name *A.rudis* with the understanding that the name refers to hybrid individuals.

#### Conservation considerations.

*Arctostaphylosnipumu* is extremely limited and restricted to the Nipomo Mesa, and, consequently of great conservation concern, especially as the subpopulations are mostly surrounded by development. Based on our estimates from satellite images, the vast majority of wild lands on the Nipomo Mesa have been converted into dwellings and agricultural fields. Fig. 5 shows the estimated land occupied by *A.nipumu* based on field surveys and satellite images. Thus, the range of *A.nipumu* consists of only 28.63 hectares distributed across 10 fragmented multi-individual occurrences, with a total population estimated to be 300–700 individuals. Similar land use change led to the near extinction of *A.franciscana*, which is now represented by a lone individual in the wild and clones of just a few genetic individuals (Gluesenkamp et al. 2010; Weiss et al. 2020). The genus *Arctostaphylos* reliably dates back to western Nevada in the Middle Miocene in a climate more similar to the California coast than present (Edwards 2004; Millar and Woolfenden 2016). Conserving the integrity of the genome of *A.nipumu* could facilitate future research testing the hypothesis that this species represents a case of paleoendemism that may provide insight into broader patterns of diversification in the genus.

We would like to emphasize that the characters used to separate *A.nipumu* from *A.rudis* are readily apparent. We were able to immediately distinguish the ca. 53 relictual *A.nipumu* individuals from the ca. 168 *A.rudis* individuals which we believe were planted as a mitigation measure to develop the Blacklake Golf Resort, as well as the restoration planting along Osage Street on the Nipomo Mesa. The ecological impact report in the Black Lake Specific Plan recommended that *Arctostaphylosrudis* be planted in parts of the Blacklake Golf Resort with low vegetation cover, not used as turf (San Luis Obispo County 1982: IV-33). Based on their burls and smooth red young bark, we propose that the majority of plants at these sites were planted cultivars originally sourced from the Burton Mesa or Point Sal; however, the origin of the nursery stock used for restoration at this site remains uncertain. Even when sound ecological considerations are implemented, mitigation and revegetation efforts such as these have often historically neglected to consider potential genetic impacts on native plants and population structure through processes such as genetic swamping, demographic swamping and outbreeding depression, among others (Hufford and Mazer 2003, Prentis et al. 2007, Byrne et al. 2011). The integrity of the genome of *A.nipumu* appears to depend on its isolation from other *Arctostaphylos* species, and these introduced plants have the potential to hybridize with *A.nipumu*. Because of their potential to hybridize, allopatry is hypothesized as an important factor maintaining the separation of *Arctostaphylos* species within the same clade (Parker et al. 2020). This stresses the importance of utilizing plants with local genotypes for propagation in restoration and mitigation projects (Maschinski and Albrecht 2017). Below is a key of *Arctostaphylos* species from San Luis Obispo and Santa Barbara Counties to aid in the identification of *A.nipumu*.

### ﻿Key

**Table:** 

1a	Burl present, plants resprouting after fire	**2**
2a	Leaves isofacial, similar in color and hairiness and with stomata on both surfaces	**3**
3a	At least some twig hairs glandular	**A.glandulosassp.glandulosa**
3b	Twig hairs nonglandular	**4**
4a	At least some twig hairs long	**A.glandulosassp.mollis**
4b	Twig hairs short	**5**
5a	Older stem bark gray, shredding	***A.rudis***
5b	Older stem bark red, smooth	**6**
6a	Endocarp fused into rough, depressed, often imperfectly symmetrical structure, nutlets requiring force to separate	**A.glandulosassp.gabrielensis**
6b	Endocarp of free or easily separable nutlets	**A.glandulosassp.cushingiana**
2b	Leaves bifacial, surfaces differing in color or hairiness, with stomata only abaxially	**7**
7a	Older stem bark grey, shredding	**8**
8a	At least some twig hairs long	**A.tomentosassp.daciticola**
8b	Twigs hairs short, tomentose only	**A.tomentosassp.tomentosa**
7b	Older stem bark red, smooth	**9**
9a	At least some twig hairs glandular	**A.crustaceassp.subcordata**
9b	All twig hairs nonglandular	**10**
10a	At least some twig hairs long	**A.crustaceassp.crustacea**
10b	All twig hairs short	**11**
11a	Leaves densely hairy abaxially	**A.crustaceassp.insulicola**
11b	Leaves glabrous to very sparsely minute pubescent abaxially	**12**
12a	Pedicels and ovary hairy	**A.crustaceassp.eastwoodiana**
12b	Pedicels and ovary glabrous to very sparsely minute pubescent	**A.crustaceassp.rosei**
1b	Burl absent, plants not resprouting after fire or resprouting ability unknown (*A.nipumu*, *A.rudis* [Point Sal])	**13**
13a	Leaves bifacial, surfaces differing in color or hairiness, with stomata only abaxially	**14**
14a	Young leaves tomentose abaxially	***A.morroensis***
14b	Young leaves glabrous to very sparsely minute pubescent abaxially	***A.insularis***
13b	Leaves isofacial, similar in color and hairiness and with stomata on both surfaces	**15**
15a	Endocarp always fused into smooth, sphere	**16**
16a	Leaves deeply lobed, auriculate to subauriculate	**17**
17a	At least some twig hairs glandular	***A.refugioensis***
17b	Twig hairs nonglandular	***A.pechoensis***
16b	Leaves unlobed to shallowly lobed, not auriculate or subauriculate	**18**
18a	Leaves glaucous	***A.glauca***
18b	Leaves bright green	**A.parryanassp.parryana**
15b	Endocarp of free to separable nutlets in at least some fruits, and when ± fused generally not a smooth sphere	**19**
19a	Older stem bark gray, shredding	**20**
20a	Leaves deeply lobed, auriculate to subauriculate	***A.osoensis***
20b	Leaves unlobed to shallowly lobed, not auriculate or subauriculate	**21**
21a	Younger stem bark smooth, red or if shredding, generally dark brown and outer bark not contrasting in color with inner bark	***A.rudis***
21b	Younger stem bark generally shredding, often gray, sometimes lighter red, then outer bark strongly contrasting in color with inner bark	***A.nipumu***
19b	Older stem bark red, smooth	**22**
22a	Bracts scale-like	**23**
23a	Plant prostrate to mounded	**A.hookerissp.hearstiorum**
23b	Plant erect	***A.pungens***
22b	Bracts leaf-like	**24**
24a	At least some twig hairs glandular	**25**
25a	Leaves deeply lobed, auriculate to subauriculate	**A.purissimassp.globosa**
25b	Leaves unlobed to shallowly lobed, not auriculate or subauriculate	***A.confertiflora***
24b	Twig hairs nonglandular	**26**
26a	Plant prostrate to mounded	***A.cruzensis***
26b	Plant erect	**27**
27a	Leaves gray, canescent	**28**
28a	Leaves deeply lobed, auriculate to subauriculate	***A.luciana***
28b	Leaves unlobed to shallowly lobed, not auriculate or subauriculate	***A.obispoensis***
27b	Leaves green	**29**
29a	Leaves unlobed to shallowly lobed, not auriculate or subauriculate	***A.pilosula***
29b	Leaves deeply lobed, auriculate to subauriculate	**30**
30a	Pedicels with moderately dense nonglandular hairs	***A.viridissima***
30b	Pedicels glabrous or with sparse nonglandular hairs	**31**
31a	Leaves ≥ 2 cm; fruit ≥ 8 cm wide; nutlets generally requiring force to separate	***A.pechoensis***
31b	Leaves ≤ 2.5 cm; fruit ≤ 8 cm wide; nutlets easily separable to free	**A.purissimassp.purissima**

## ﻿Conclusion

Hybridization plays a key role in the evolutionary dynamics of *Arctostaphylos* species. Differences in ploidy and genetic distance impose incomplete reproductive isolation between species that occur in sympatry. *A.purissima* and *A.crustacea* remain genetically and morphologically distinct in sympatry, while *A.nipumu* is replaced by the hybrid *A.rudis* when it occurs with *A.purissima* and/or *A.crustacea*. *A.nipumu* is able to persist as a distinct species apparently because of its geographic isolation from other *Arctostaphylos* on the Nipomo Mesa. Its habitat is fragmented and should be considered as a high priority for conservation efforts. Future work should investigate its demographic history to better understand the patterns that led to its isolation and the circumstances that led to its hypothesized extinction by hybridization south of the Santa Maria River.

## Supplementary Material

XML Treatment for Arctostaphylos nipumu

## ﻿Additional information

### Author ORCIDs

Tito Abbo https://orcid.org/0009-0004-0110-8336

Morgan A. Stickro https://orcid.org/0000-0002-3177-7379

Alexander Krohn https://orcid.org/0000-0002-3152-2915

V. Thomas Parker https://orcid.org/0000-0002-6149-7140

Michael C. Vasey https://orcid.org/0000-0002-5036-3307

Amy Litt https://orcid.org/0000-0001-6709-8696

### Data availability

All of the data that support the findings of this study are available in the main text or

Supplementary Information. Raw DNA sequences are pubically available in the NCBI Sequence Read Archive (SRA) under the project number PRJNA1208541.

## Supplementary materials

Supplementary material 1 Samples/collections used in study Data type xlsx Explanation note Specimens are identified in the first column by the collector’s last name followed by collector’s number. The individual code in column 2 refers to the one to two letter, single number code used to refer to samples in figures and/or the main text. The taxon column gives our determination of the specimen/sample’s taxonomic identity (*A.* = *Arctostaphylos*; ssp.= subspecies). The date column refers to the date the specimen/sample was collected. The Latitude and Longitude columns are the GPS coordinates of where specimen/sample was obtained in decimal degrees. The Herbaria column gives the herbaria codes of where specimens (and if collected their duplicates) are housed. The type column indicates whether the specimen is a type (No = not a type, Holo = Holotype, Iso = Isotype, Para = Paratype). The CCH2 links columns gives a link to additional information about and an image of the specimen stored on the CCH2 data portal; specimens Abbo 147–156 are not available on CCH2 at the time of the manuscript’s submission but will be added to the portal soon. https://binary.pensoft.net/file/1221514This dataset is made available under the Open Database License (http://opendatacommons.org/licenses/odbl/1.0/). The Open Database License (ODbL) is a license agreement intended to allow users to freely share, modify, and use this Dataset while maintaining this same freedom for others, provided that the original source and author(s) are credited.Tito Abbo, Morgan A. Stickrod, Alexander Krohn, V. Thomas Parker, Michael C. Vasey, William Waycott, Amy Litt

Supplementary material 2 Comparison of principal component analyses Data type png Explanation note Comparison of principal component analyses (PCA) of ddRADseq data with SNPs present in at least 80% of individuals (minimum coverage = 0.8). The figure was generated using the IPYRAD analysis tools (IPYRAD version 0.9.95) and edited in Inkscape version 1.1.2. Solid circles are the results of the PCA for a random draw of uncorrelated SNPs for each sample, and the transparent circles are the results of 49 additional PCAs for distinct, independent random draws of uncorrelated SNPs. Samples from the Nipomo Mesa (green), Point Sal (yellow), and Burton Mesa and Harris grade (blue) are color-coded by population, and samples of *Arctostaphylospurissima* (orange), and *A.crustacea* (purple) from control/nonfocal populations are color-coded by taxonomic identity. In each tile, PC0 is given in the x axis and PC1 in the y axis. The amount of variation each axis explains is indicated on the axis (A. PC0 30.1%, PC1 6.1%; B. PC0 16.8%, PC1 6.5%; C. PC0 17.7%, PC1 4.7%; D. PC0 32.6%, PC1 6.1%; E. PC0 15.1%, PC1 5.2%; F. PC0 15.6%, PC1 5.7%). A–C. The raw sequence data was processed in IPYRAD with the maximum alleles allowed in the consensus sequence set to 4 (max_alleles_consens = 4). D–F. The raw sequence data was processed in IPYRAD with max_alleles_consens = 2. A. & D. All 54 samples were included, and missing data was imputed using K-means imputation at K = 4. B. & E. All 54 samples were included, and no missing data imputation was used. C. & F. Samples I8, CZ6, DA3, DC5 with higher missing data were omitted from the analysis (total sample size = 50), and no missing data imputation was used (see Suppl. material 1 for the taxonomic identities and other details about these samples). https://binary.pensoft.net/file/1221515This dataset is made available under the Open Database License (http://opendatacommons.org/licenses/odbl/1.0/). The Open Database License (ODbL) is a license agreement intended to allow users to freely share, modify, and use this Dataset while maintaining this same freedom for others, provided that the original source and author(s) are credited.Tito Abbo, Morgan A. Stickrod, Alexander Krohn, V. Thomas Parker, Michael C. Vasey, William Waycott, Amy Litt

Supplementary material 3 Structure plots/stacked bar graphs Data type png Explanation note Structure plots/stacked bar graphs of the 3^rd^ of 3 replicates for each of K ancestral groups from K = 1 (top) to K = 10 (bottom). Structure was run using STRUCTURE_THREADER version 1.3.10 for 50,000 MCMC generations with the first 10,000 discarded as burn-in. The individual bar graphs were generated using STRUCTURE_THREADER and were combined and edited in Inkscape version 1.1.2. Each individual sample is represented by a unique 1–2 letter and single number code given at the bottom of the x axis. The y axis indicates the genotype percentage of the K ancestral groups. The two most prominent ancestral groups are drawn in green and orange, and for K values > 2, less prominent ancestral groups are arbitrarily represented by different colors but these occur at such low percentages that they are often not visible in the figure. https://binary.pensoft.net/file/1221516This dataset is made available under the Open Database License (http://opendatacommons.org/licenses/odbl/1.0/). The Open Database License (ODbL) is a license agreement intended to allow users to freely share, modify, and use this Dataset while maintaining this same freedom for others, provided that the original source and author(s) are credited.Tito Abbo, Morgan A. Stickrod, Alexander Krohn, V. Thomas Parker, Michael C. Vasey, William Waycott, Amy Litt
