# Supplementary figures and images for: ﻿Investigating a hybrid mixed population leads to recognizing a new species of Arctostaphylos (Ericaceae)
Source: PhytoKeys. 2025 Jan 16;251:119–42. doi: 10.3897/phytokeys.251.139172 (PMC11758095; doi:10.3897/phytokeys.251.139172)

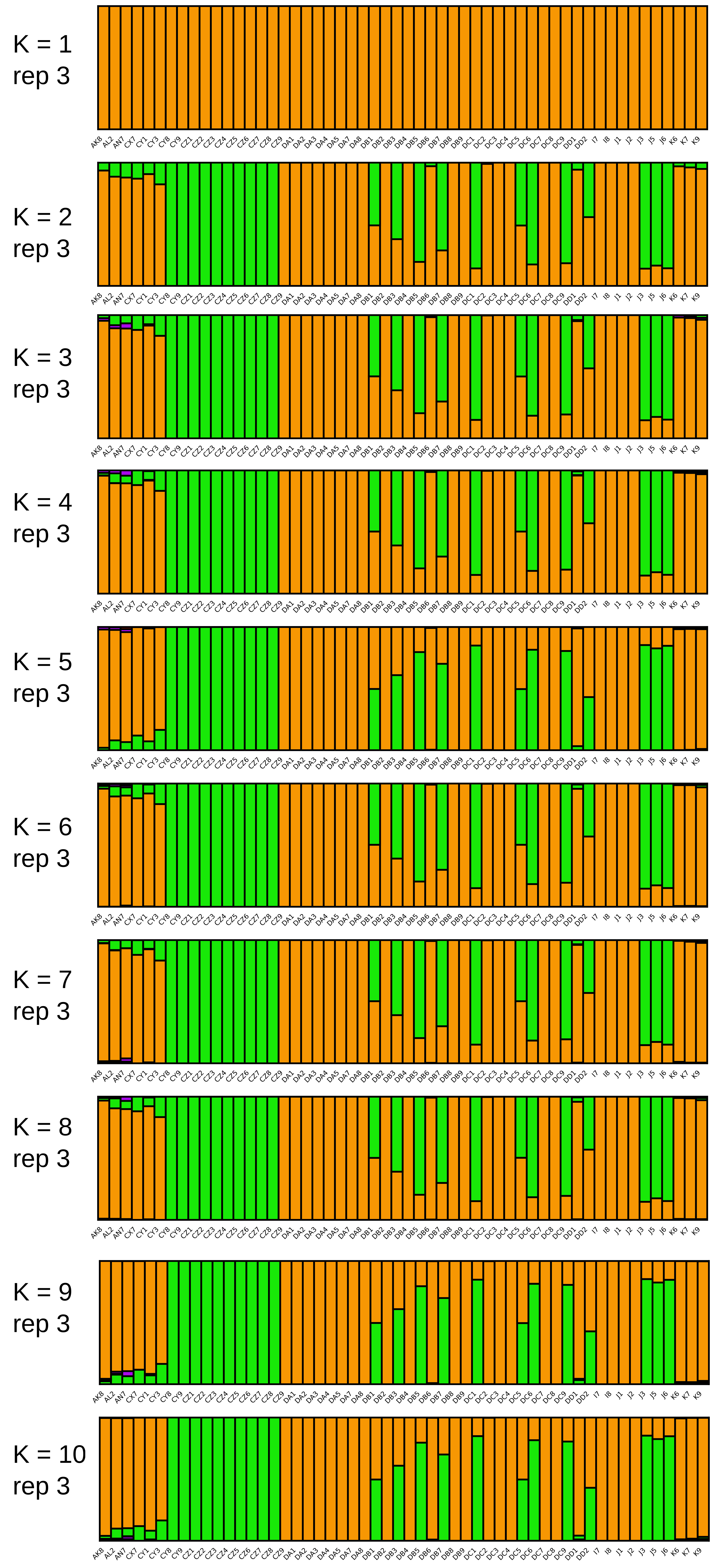

Supplement: Supplementary material 3 — Structure plots/stacked bar graphs [file phytokeys-251-119_article-139172__-s003.png]
